# Antibacterial Activity of Defatted and Nondefatted Methanolic Extracts of *Aframomum melegueta* K. Schum. against Multidrug-Resistant Bacteria of Clinical Importance

**DOI:** 10.1155/2020/4808432

**Published:** 2020-08-06

**Authors:** Olufunmiso Olusola Olajuyigbe, Otunola Adedayo, Roger Murugas Coopoosamy

**Affiliations:** ^1^Department of Microbiology, Babcock University, PMB 4005, Ilisan-Remo, Ogun State, Nigeria; ^2^Department of Nature Conservation, Mangosuthu University of Technology, Durban, KwaZulu-Natal, South Africa; ^3^Department of Microbiology, Kwara State University, Ilorin, Kwara State, Nigeria

## Abstract

The antibacterial activity of the extracts of *Aframomum melegueta* including *n*-hexane extract (NHE), nondefatted methanol extract (NDME), and defatted methanol extract (DME) was investigated in this study. The NHE exhibited no antibacterial activity. The DME showed higher antibacterial activity than the NDME against the different isolates. At the highest concentration of 10 mg/mL in agar diffusion, NDME produced inhibition zones ranging from 11 to 29 mm against the microorganisms while DME produced inhibition zones ranging from 20 to 40 mm with the concentration of 10 mg/mL against the microorganisms. 0.1 mg/mL of the DME produced inhibition zones ranging between 12 and 14 mm in *Aeromonas hydrophila* ATCC 35654 and *Pseudomonas aeruginosa* ATCC 15442, respectively, while none of the isolates were inhibited by the NDME at a concentration of 1 mg/mL or less. In the agar dilution assay, the MICs of the NDME and DME ranged between 0.31 and 10 mg/mL, but more isolates were inhibited at 0.31 mg/mL of DME than those in NDME. In macrobroth assay, the MICs of the NDME ranged between 0.15 and 5.0 mg/mL and the MBCs ranged between 0.63 and 5.0 mg/mL, and the MICs of the DME ranged between 0.08 and 5.0 mg/mL and the MBCs were between 0.31 and 5.0 mg/mL. This study indicated that DME was more active with higher antibacterial activity than the NDME of this plant, and extracting the fatty portion of plant materials prior susceptibility testing would allow plant extracts to be more effective as well as justifying the use of *Aframomum melegueta* in traditional medicine for the treatment of bacterial infections.

## 1. Introduction

Medicinal plants have been used for centuries as remedies for human diseases because they contain components of therapeutics value. Recently, some higher plants' phytochemicals have been exploited as antimicrobials because they are biodegradable and not deleterious to human health [1] and highly acceptable [2]. The plant kingdom, for a long time, has, therefore, served as a prolific source of helpful drugs, food additives, flavoring agents, binders, and lubricants. It was estimated that about 25% of all prescribed medicines today are substances derived from plants [3]. While the acceptance of traditional medicine as an alternate form of health care encouraged researchers to further investigate antimicrobial activity of many plants, the use of herbal medicine, in the treatment and prevention of diseases, predating the introduction of antibiotics and other modern drugs [4], has continued to attract more attention to scientists worldwide [5], and more than 80% of the population in developing countries of the world depend on traditional herbal medicine [6].

In addition to this, emergence, persistence, and spread of multidrug-resistant (MDR) bacteria or “superbugs” [7] have posed a serious global threat of growing concern to treatment of infections. The “superbugs” denoting microorganisms with higher morbidity and mortality rate have increased due to several mutations that resulted in resistance to various classes of antibiotics. This is, on the other hand, due to the use and misuse of antimicrobials, enhanced global migration, increased use of antibiotics in clinics and animal production, selection pressure, poor sanitation, wildlife spread, and poor sewerage disposal system [8, 9]. In medical arena, the increased antibiotic resistance has become a major threat to public health as effectiveness of antimicrobial treatments has reduced drastically while morbidity, mortality, and health care cost have increased significantly. While the death of the golden era of antibiotics resulted from the inability of researchers to maintain the pace at which antibiotics are discovered in the face of emerging resistant pathogens [10] and persistent failure to develop or discover new antibiotics and nonjudicious use of antibiotics are the predisposing factors associated with the emergence of antibiotic resistance [11], there is the need to search for alternative means of treating microbial infections globally as success in treating infectious diseases depends on the discovery of new and more antimicrobial compounds [12].

*Aframomum melegueta* K. Schum. is a species in the Zingiberaceae family. It is a spice commonly known as ‘grains of paradise' [13], melegueta pepper, alligator pepper, guinea grains, or guinea pepper. It is a herbaceous perennial plant native to swampy habitats along the West African coast and an important cash crop in the southern part of Ethiopia [14]. Its trumpet-shaped purple flowers developing into 5 to 7 cm long pods containing numerous small, reddish-brown seeds. The average leaves are usually 35 cm in length and 15 cm wide. While the flowers are described as “handsome” aromatic with an orange colored lip and rich pinkish-orange upper part and the fruits contain numerous small golden red-brown seeds [15], the seed has a pungent odor, peppery, and slightly bitter taste [16] caused by aromatic ketones named 1-4-hydroxy-3-methoxyphenyl-decan-3-one [17]. The seeds have antimicrobial effects [18] and are used as a remedy for diarrhea, stomachache, rheumatism, inflammatory conditions, and postpartum hemorrhage [18–20].

Although various species of the *Aframomum* plants have been used as antidiarrhoea, anti-inflammatory, laxatives, for stomachache management, and as a tonic for sexual stimulation [21], El-Halawany et al. [22] indicated that *Aframomum* plants have antiulcer, antiplasmodial, antinociceptive, antimicrobial, and anticancer properties. The phytochemicals obtained from the seeds of *A*. *melegueta* have been used for years in the treatment of infectious diseases, and its grains possess active ingredients that may be exploited for local development of antimicrobials [23]. The antimicrobial activities have been attributed to the presence of phenols and phenolic compounds in its seeds [24] and terpenoids such as aframodial [25]. Amadi and Wu, [26] reported that the potentials of phenols and terpenoids isolated from *Aframomum* plants have been well documented. Since the seed extracts exhibited antiseptic and bactericidal potentials locally, they play significant roles in treating wounds and in preventing infections [27]. Though there is dearth of information comparing antibacterial activity of fatted and defatted extracts of this plant, this study aimed at investigating the antibacterial activities of fatted and defatted methanolic extracts of *A*. *melegueta* seeds with those of some selected antibiotics widely used against bacterial infections.

## 2. Materials and Methods

### 2.1. Collection of Plant Material

Fresh fruits of *Aframomum melegueta* were purchased from a local market in Osun state, Nigeria. The fruits were sun-dried and authenticated at the Department of Basic Sciences, Babcock University, Ilisan Remo, Ogun State, Nigeria.

### 2.2. Test Microorganisms

Ten American Type Culture Collection (ATCC) pure cultures of bacteria were obtained from the Department of Microbiology and Biochemistry, University of Fort Hare, South Africa. The test organisms used include *Micrococcus luteus*, *Pseudomonas aeruginosa* ATCC 15442, *Bacillus subtilis* KZN, *Plesiomonas shigelloides* ATCC 51903, *Aeromonas hydrophila* ATCC 35654, *Staphylococcus aureus* NCTC 6571, *Escherichia coli* ATCC 25922, *Klebsiella pneumoniae* ATCC 10031, *Pseudomonas aeruginosa* ATCC 19582, and *Klebsiella pneumoniae* ATCC 4352. The isolates were maintained on nutrient agar at 4°C.

### 2.3. Extract Preparation

The seeds of sun-dried *A. melegueta* were collected and further air-dried at room temperature before being pulverized in a mill (Christy Lab Mill, Christy and Norris Ltd; Process Engineers, Chelmsford, England) and stored in a sterile air-tight container for further use. To prepare *n*-hexane extract (NHE), 400 g of the pulverized seeds was steeped in 1500 mL of *n*-hexane for 72 h with shaking (Stuart Scientific Orbital Shaker, UK). To prepare nondefatted methanol extract (NDME), 150 g of the seed powder was steeped in 1000 mL of methanol for 72 h. To prepare defatted methanol extract (DME), the powdered seed material soaked in *n*-hexane was extracted and air-dried for three days before being soaked again in methanol for 72 h. The steeped seed materials in all the solvent were extracted after the soaking period for two other consecutive times. The extracts from each preparation were combined, filtered through Whatman No. 1 filter paper and concentrated under reduced pressure at 40°C using a rotary evaporator (Laborota 4000–efficient, Heidolph, Germany). Each of the crude extracts collected was dried at room temperature to a constant weight. The resulting crude extracts were stored in desiccators containing silica beads to remove moisture from the extract until used for assay. The extracts were resuspended in each of the extracting solvents to yield a 40 mg/mL stock solution from which different concentrations were prepared for analysis.

### 2.4. Antibacterial Activities of the Standard Antibiotics and Those of the Extracts by Agar Diffusion Techniques

Each of the isolates was prepared by the colony suspension method [28]. The density of each strain's suspension was adjusted to equal that of the 0.5 McFarland standards by adding sterile distilled water to give a resultant concentration of 1 × 10^7^ cfu/mL. The antibacterial activity was determined using the agar diffusion method according to the modified Kirby–Bauer diffusion technique [29, 30] by swabbing the Mueller Hinton agar (MHA) (Oxoids UK) plates with the resultant standardized overnight culture of each test isolate. Multidiscs containing different antibiotics including ofloxacin (5 *µ*g), ciprofloxacin (5 *µ*g), gentamicin (10 *µ*g), cefuroxime (30 *µ*g), ceftazidime (30 *µ*g), ampicillin (10 *µ*g), augumentin (30 *µ*g), and nitrofurantoin (300 *µ*g) were aseptically placed on the inoculated agar plates and incubated at 37°C for 24 h. After 24 h of incubation, the plates were examined for zones of inhibition [31]. The diameter of the inhibition zones produced by the extracts were measured and interpreted using the Clinical and Laboratory Standard Institute zone diameter interpretative standards [32].

To determine the antibacterial activities of the different extracts, plates were prepared according to the modified Kirby–Bauer by swabbing the Mueller Hinton agar (MHA) (Oxoids UK) plates with the resultant standardized overnight culture of each test isolate before wells were bored into the sets of the inoculated agar medium with heat-sterilized 6 mm cork borer. The wells were filled with 100 *µ*L of different concentrations (0.1, 10, and 100 mg/mL) prepared for each of the extracts taking care not to allow spillage of the solutions onto the surface of the agar. The plates were allowed to stand for at least 30 min before being incubated at 37°C for 24 h [33]. After 24 h of incubation, the plates were examined for zones of inhibition [31]. The diameter of the inhibition zones produced by the extracts were measured and interpreted using the Clinical and Laboratory Standard Institute zone diameter interpretative standards [32].

### 2.5. Determination of the Minimum Inhibitory Concentrations (MICs) of the Different Extracts by Agar Dilution Techniques

To determine the antibacterial activity of the extract by the agar dilution method as described by Afolayan and Meyer [34], different concentrations of the extract, 0.1 mg/mL, 1.0 mg/mL, and 10 mg/mL, were prepared in molten Mueller Hinton agar maintained in a water bath at 50°C. A loopful of each of the standardized bacterial cultures was aseptically inoculated into the solidified agar by stabbing. Two Mueller Hinton agar plates containing 5% methanol representing the final methanol concentration in the test plates without the extract served as negative controls. Another two blank plate containing only Mueller Hinton agar served as negative controls. Plates were incubated at 37°C for 24 h. Each test was done in duplicate, and any test agar plate lacking visible growth at the point of stabbing was considered the minimum inhibitory concentration of each of the extracts

### 2.6. Determination of Minimum Inhibitory Concentrations (MICs) of the Different Extracts by Macrobroth Dilution

The minimum inhibitory concentrations (MICs) for the two extracts were determined in duplicate by the macrobroth dilution method in Mueller Hinton broth according to the Clinical Laboratory Standardization Institute (CLSI) [35]. To determine the MICs of each extract, different concentrations (0.02, 0.04, 0.08, 0.16, 0.3125, 0.625, 1.25, 2.5, 5.0, and 10.0 mg/mL) of each of the extracts were prepared by serial dilution in double-strength Mueller Hinton broth. After the serial dilution, each tube was inoculated with 100 *µ*L of each of the bacterial strains and incubated at 37°C for 24 h. Blank Mueller Hinton broth was used as negative control. Each assay was performed two times. The MIC was defined as the lowest dilution that showed no bacterial growth in the Mueller Hinton broth.

### 2.7. Determination of the Minimum Bactericidal Concentrations (MBCs) of the Different Extracts

The minimum bactericidal concentrations (MBCs) were identified by determining the lowest concentration of antibacterial agent that reduces the viability of the initial bacterial inoculum by ≥99.9%. Here, antibiotic-free nutrient agar plates were inoculated with one loopful of culture taken from each of the first two broth cultures that showed no growth and the first growth-containing tube in the MIC tubes. The MBC plates were incubated at 37°C for 24 h. After the incubation periods, the lowest concentration of each of the extracts that did not produce bacterial growth on the solid medium was regarded as their MBC values. This observation was matched with the MIC test tubes that did not show evidence of growth after 48 h of incubation.

## 3. Results

With the exception of ofloxacin and ciprofloxacin to which all the isolates were susceptible and gentamicin to which two of the isolates were resistant, cefuroxime, ceftazidime, ampicillin, and augumentin were ineffective while nitrofurantoin was able to inhibit the growth of five of the isolates. While the ofloxacin produced inhibition zones ranging between 15 and 30 mm, ciprofloxacin produced inhibition zones ranging between 16 and 25 mm as shown in Table 1.

The antibacterial activities of the three extracts including *n*-hexane extract (NHE), nondefatted methanol extract (NDME), and defatted methanol extract (DME) are shown in Table 2. The NHE exhibited no antibacterial activity. This showed that the fatty portion of the plant extract had no antibacterial activity. The NDME, on the contrary, was able to inhibit the test isolates at an average level as compared with the DME that showed high potency and a better antibacterial activity against the different isolates. At the highest concentration of 10 mg/mL, NDME produced inhibition zones ranging from 11 to 29 mm, while the isolates were not susceptible at a concentration of 1 mg/mL or less. While DME produced inhibition zones ranging from 20 to 40 mm at the highest concentration of 10 mg/mL, 1 mg/mL of this extract produced inhibition zones ranging from 14 to 20 mm with the exception of *S*. *aureus* NCTC 6571 and *Klebsiella pneumonia*e ATCC 4352 that were resistant. Contrariwise, 0.1 mg/mL of the DME was able to produce inhibition zones ranging between 12 and 14 mm in *Aeromonas hydrophila* ATCC 35654 and *Pseudomonas aeruginosa* ATCC 15442, respectively. Other isolates were not susceptible to this extract at 0.1 mg/mL.

In Table 3, the susceptibility of the bacterial isolates was compared using agar dilution and macrobroth dilution assays based on the fact that the extracts were thoroughly mixed in the respective media while the isolates had a direct contact with the extract in the macrobroth dilution. The agar dilution assay showed that NDME and DME inhibited the isolates at minimum inhibitory concentrations ranging from 0.31 to 10 mg/mL. For the NDME, *Pseudomonas aeruginosa* ATCC 15442 was inhibited at the MIC of 0.15 mg/mL, *Micrococcus luteus*, *Escherichia coli* ATCC 25922, *Aeromonas hydrophila* ATCC 35654, and *Plesiomonas shigelloides* ATCC 51903 had their MICs at 0.31 mg/mL, *Klebsiella pneumoniae* ATCC 4352 had its MIC at 0.63 mg/mL, while *Bacillus subtilis* KZN, *Staphylococcus aureus* NCTC 6571, *Klebsiella pneumoniae* ATCC 10031, and *Pseudomonas aeruginosa* ATCC 19582 had their MICs at 5.0 mg/mL. For the DME, *Klebsiella pneumoniae* ATCC 4352, *Pseudomonas aeruginosa* ATCC 15442, *Aeromonas hydrophila* ATCC 35654, *Micrococcus luteus*, and *Plesiomonas shigelloides* ATCC 51903 had 0.31 mg/mL as their MICs, *Escherichia coli* ATCC 25922 had an MICs of 0.63 mg/mL, *Pseudomonas aeruginosa* ATCC 19582, *Klebsiella pneumoniae* ATCC 10031, and *Staphylococcus aureus* NCTC 6571 had their 5.0 mg/mL as their MICs, while *Bacillus subtilis* KZN had MICs of 10 mg/mL. In comparison, the MICs and MBCs were obtained from the macrobroth dilution. From the macrobroth dilution, while the MICs of the NDME ranged between 0.15 and 5.0 mg/mL and the MBCs ranged between 0.63 and 5.0 mg/mL, the MICs of the DME ranged between 0.08 and 5.0 mg/mL and the MBCs were between 0.31 and 5.0 mg/mL. The DMEs of the *Aframomum melegueta* was 50% higher in antibacterial activity than those of the NDME.

## 4. Discussion

Plants have a long history of antibiotic usage for the cure of disease caused by microorganisms. Many extracts from different plants have been tested for their antimicrobial activities, but there are still many plants requiring more careful investigation to reveal their hidden potential(s). While increasingly difficult socioeconomic situations and unavailability of modern medicine at affordable prices have forced tropical and developing countries, recording high rate of infectious diseases, to rely on traditional medicines [36, 37], antimicrobial agents from medicinal plants are increasingly showing their potentials to cure invasive infections [38, 39]. Therefore, the acceptance of traditional medicine as an alternative form of health care and the development of microbial resistance to the available antibiotics has led scientists to investigate the antimicrobial activity of medicinal plants [40]. The medicinal plants are, thus, recognized as a source of new drugs and complementary medicines, neutraceuticals, pharmaceuticals, and intermediate chemicals for synthetic drugs and their versatile applications [41–43].

In this study, the antibacterial activity of the two different extracts against the various bacterial isolates showed the scientific validity of *Aframomum melegueta* being used traditionally as a medicine. The results indicated that defatted methanolic extract (DME) was more active with higher antibacterial activity than the nondefatted methanolic extract (NDME) of this plant. The activity of the NDME at the highest concentration of 10 mg/mL was equivalent to the activity of the DME at a concentration of 1 mg/mL. Thus, DME inhibited the bacterial isolates at lower concentrations compared with what were obtained from the NDME. While their antibacterial activities may be attributed to the presence of 6-paragol and 6-shogoal [17] as well as soluble phenolic and polyphenolic compounds in the seeds [44], intercalation of the extract with deoxyribonucleic acid (DNA) [45], and disturbance of the permeability barrier of the bacterial membrane structure [46]. Balakumar et al. [47] suggested that the extract components cross the cell membrane, interact with the enzymes and proteins of the membrane, and produce a flux of protons towards the cell exterior to induce changes and the ultimate death of the cells. Pandian et al. [48] and Srinivasan [49] indicated that the bacterial cell death could have resulted from the binding of epicatechin and catechin to positively charged lipid bilayer of Gram-positive bacteria, inhibition of metabolic enzymes, and interference with the synthesis of certain amino acids necessary for bacterial growth. Olajuyigbe et al. [50] also reported that the cell death from the bacteria treated with the extracts could have resulted from ultrastructural changes and breakdown of elemental components in the bacteria into different elements and leaked lipid and protein from the bacteria. Olajuyigbe et al. [51] further reported that the cell death resulted from the ability of the extracts to produce reactive oxygen species with deleterious effects. Since the bioactivity of extracts depends on the concentration used [52], the ineffectiveness of the extracts at lower concentrations may be attributed to the presence of lesser amounts of the antimicrobial compounds in the extracts.

Though NDME and DME, respectively, inhibited 60% of the isolates at concentrations ranging between 0.08 and 0.63 mg/mL, DME showed a better antibacterial activity. This is contrary to minimum inhibitory concentration of 31.25 mg/mL against *Bacillus cereus* [53] and that of 1.51 mg/mL against *Enterococcus aerogenes*, *Staphylococcus aureus*, and *Proteus mirabilis* [54] in the previous studies with *A. melegueta*. The difference in the antibacterial activity of NDME and DME could be attributed to the inhibitory effect of the fatty portion of the plant material. Since *n*-hexane extract showed no antibacterial activity and the antibacterial activity of the NDME was lesser than that obtained from the DME, the interaction and presence of the fatty portion of the plant could have resulted in antagonistic interactions with phytochemicals having significant antibacterial activities.

Though there is a dearth of information indicating the effects of fatty portion of plant material on the antibacterial activities of varied phytochemicals, there have been several reports on the antibacterial activities of *n*-hexane extracts (NHEs) of some other plants [55–57]. While the NHE of *A*. *melegueta* showed no antibacterial activity against the selected bacterial strains, the antibacterial activities of NDME and DME may be considered significant as Rios and Recio [58] suggested that extracts with MICs of 0.1 mg/mL have significant antibacterial activities but those with MIC greater than 1 mg/mL should be avoided. Fabry et al. [59] defined active crude extracts as those having MIC values< 8 mg/mL. Hence, since lower MIC and MBC values indicate higher efficacy [60] and phytochemicals are routinely classified as antimicrobials when susceptibility tests had MICs in the range of 0.1–1.0 mg/mL [61], the MICs and MBCs of less than 1 mg/mL, in this study, were considered to be of good activity for both NDME and DME.

## 5. Conclusions

In conclusion, many medicinal plant extracts exert their antibacterial effects through the additive or synergistic action of several chemical compounds acting at single or multiple target sites associated with a physiological process. This study shows that extracting the fatty portion of plant materials with *n*-hexane prior to susceptibility testing could allow the effectiveness of the extracts to be brought to the fore since defatted extract was significantly more active than the nondefatted extract and justify the use of *Aframomum melegueta* in traditional medicine for the treatment of bacterial infections.

## Figures and Tables

**Table 1:** Bacterial susceptibility to different standard antibiotic disks.

	Average inhibition zones (±1.00) produced by different standard antibiotics							
Organisms used	OFL	CPR	GEN	CFR	CFZ	AMP	NIT	AUG
	(5 *µ*g)	(5 *µ*g)	(10 *µ*g)	(30 *µ*g)	(30 *µ*g)	(10 *µ*g)	(300 *µ*g)	(30 *µ*g)
	(mm)							

*Micrococcus luteus*	15 ± 1.00	16 ± 1.00	16 ± 1.00	6 ± 1.00	6 ± 1.00	6 ± 1.00	15 ± 1.00	6 ± 1.00
*Bacillus subtilis* KZN	26 ± 1.00	24 ± 1.00	25 ± 1.00	6 ± 1.00	6 ± 1.00	6 ± 1.00	27 ± 1.00	6 ± 1.00
*Escherichia coli* ATCC 25922	20 ± 1.00	20 ± 1.00	10 ± 1.00	6 ± 1.00	6 ± 1.00	6 ± 1.00	6 ± 1.00	6 ± 1.00
*Staphylococcus aureus* NCTC 6571	28 ± 1.00	25 ± 1.00	14 ± 1.00	21 ± 1.00	6 ± 1.00	6 ± 1.00	6 ± 1.00	6 ± 1.00
*Klebsiella pneumoniae* ATCC 4352	25 ± 1.00	25 ± 1.00	15 ± 1.00	6 ± 1.00	6 ± 1.00	6 ± 1.00	13 ± 1.00	6 ± 1.00
*Aeromonas hydrophila* ATCC 35654	15 ± 1.00	25 ± 1.00	15 ± 1.00	6 ± 1.00	6 ± 1.00	6 ± 1.00	15 ± 1.00	6 ± 1.00
*Klebsiella pneumoniae* ATCC 10031	25 ± 1.00	25 ± 1.00	13 ± 1.00	6 ± 1.00	6 ± 1.00	6 ± 1.00	6 ± 1.00	6 ± 1.00
*Plesiomonas shigelloides* ATCC 51903	15 ± 1.00	16 ± 1.00	6 ± 1.00	6 ± 1.00	6 ± 1.00	6 ± 1.00	6 ± 1.00	6 ± 1.00
*Pseudomonas aeruginosa* ATCC 15442	15 ± 1.00	14 ± 1.00	6 ± 1.00	6 ± 1.00	6 ± 1.00	6 ± 1.00	6 ± 1.00	6 ± 1.00
*Pseudomonas aeruginosa* ATCC 19582	30 ± 1.00	25 ± 1.00	15 ± 1.00	6 ± 1.00	6 ± 1.00	6 ± 1.00	15 ± 1.00	6 ± 1.00

Ofl = ofloxacin; Cpr = ciprofloxacin; Gen = gentamicin; Cfr = cefuroxime; Cfz = ceftazidime; Amp = ampicillin; Aug = augumentin; Nit = nitrofurantoin.

**Table 2:** Antibacterial activity of the different extracts of *Aframomum melegueta* using agar well diffusion assay.

	Average inhibition zones (±1.0 mm) produced by the different extracts of *A*. *melegueta*								
	NHE			NDME			DME		
Organisms used	10	1	0.1	10	1	0.1	10	1	0.1
	(mg/mL)								

*Micrococcus luteus*	6 ± 1.00	6 ± 1.00	6 ± 1.00	19 ± 1.00	6 ± 1.00	6 ± 1.00	25 ± 1.00	18 ± 1.00	6 ± 1.00
*Bacillus subtilis* KZN	6 ± 1.00	6 ± 1.00	6 ± 1.00	29 ± 1.00	6 ± 1.00	6 ± 1.00	40 ± 1.00	15 ± 1.00	6 ± 1.00
*Escherichia coli* ATCC 25922	6 ± 1.00	6 ± 1.00	6 ± 1.00	14 ± 1.00	6 ± 1.00	6 ± 1.00	29 ± 1.00	14 ± 1.00	6 ± 1.00
*Staphylococcus aureus* NCTC 6571	6 ± 1.00	6 ± 1.00	6 ± 1.00	19 ± 1.00	6 ± 1.00	6 ± 1.00	23 ± 1.00	6 ± 1.00	6 ± 1.00
*Klebsiella pneumoniae* ATCC 4352	6 ± 1.00	6 ± 1.00	6 ± 1.00	14 ± 1.00	6 ± 1.00	6 ± 1.00	20 ± 1.00	6 ± 1.00	6 ± 1.00
*Aeromonas hydrophila* ATCC 35654	6 ± 1.00	6 ± 1.00	6 ± 1.00	20 ± 1.00	6 ± 1.00	6 ± 1.00	25 ± 1.00	16 ± 1.00	12 ± 1.00
*Klebsiella pneumoniae* ATCC 10031	6 ± 1.00	6 ± 1.00	6 ± 1.00	14 ± 1.00	6 ± 1.00	6 ± 1.00	20 ± 1.00	6 ± 1.00	6 ± 1.00
*Plesiomonas shigelloides* ATCC 51903	6 ± 1.00	6 ± 1.00	6 ± 1.00	11 ± 1.00	6 ± 1.00	6 ± 1.00	24 ± 1.00	6 ± 1.00	6 ± 1.00
*Pseudomonas aeruginosa* ATCC 15442	6 ± 1.00	6 ± 1.00	6 ± 1.00	20 ± 1.00	6 ± 1.00	6 ± 1.00	30 ± 1.00	20 ± 1.00	14 ± 1.00
*Pseudomonas aeruginosa* ATCC 19582	6 ± 1.00	6 ± 1.00	6 ± 1.00	16 ± 1.00	6 ± 1.00	6 ± 1.00	30 ± 1.00	15 ± 1.00	6 ± 1.00

NHE = *n*-hexane extract; NDME = nondefatted methanol extract; DME = defatted methanol extract.

**Table 3:** Antibacterial activities of NDME and DME of *Aframomum melegueta* as determined by both agar and macrobroth dilution techniques.

	Agar dilution		Broth dilution			
	NDME	DME	NDME		DME	
Organisms used	MIC	MIC	MIC	MBC	MIC	MBC
	(mg/mL)					

*Micrococcus luteus*	0.63	0.31	0.31	0.63	0.15	0.31
*Bacillus subtilis* KZN	10.0	10	5.0	10.0	5.0	5.0
*Escherichia coli* ATCC 25922	0.63	0.63	0.31	0.63	0.31	0.31
*Staphylococcus aureus* NCTC 6571	10.0	5.0	5.0	10.0	2.5	5.0
*Klebsiella pneumoniae* ATCC 4352	1.25	0.31	0.63	10.0	0.31	0.31
*Aeromonas hydrophila* ATCC 35654	0.63	0.31	0.31	1.25	0.15	0.31
*Klebsiella pneumoniae* ATCC 10031	10.0	5.0	5.0	1.25	2.5	5.0
*Plesiomonas shigelloides* ATCC 51903	0.63	0.31	0.31	0.63	0.15	0.31
*Pseudomonas aeruginosa* ATCC 15442	0.31	0.31	0.15	0.63	0.08	0.15
*Pseudomonas aeruginosa* ATCC 19582	10.0	5.0	5.0	10.0	2.5	5.0

## Data Availability

The data used to support the findings of this study are included within the article
